# Piezo1 activation induces relaxation of the pudendal artery and corpus cavernosum

**DOI:** 10.3389/fphys.2023.998951

**Published:** 2023-02-10

**Authors:** Vanessa Dela Justina, Raiany Alves de Freitas, Olufunke O. Arishe, Fernanda R. Giachini, R. Clinton Webb, Fernanda Priviero

**Affiliations:** ^1^ Graduate Program in Biological Sciences, Federal University of Goias, Goiânia, Goias, Brazil; ^2^ Department of Cell Biology and Anatomy—School of Medicine, University of South Carolina, Columbia, SC, United States; ^3^ Cardiovascular Translational Research Center—School of Medicine, University of South Carolina, Columbia, SC, United States; ^4^ Institute of Biological Sciences and Health, Federal University of Mato Grosso, Barra do Garças, Mato Grosso, Brazil

**Keywords:** Piezo1 channel, mechanosensory, shear-stress, vascular function, cavernosal function

## Abstract

Piezo1 channel is a sensor for shear-stress in the vasculature. Piezo1 activation induces vasodilation, and its deficiency contributes to vascular disorders, such as hypertension. In this study, we aimed **t**o determine whether Piezo1 channel has a functional role in the dilation of pudendal arteries and corpus cavernosum (CC). For this, male Wistar rats were used, and the relaxation of the pudendal artery and CC was obtained using the Piezo1 activator, Yoda1, in the presence and absence of Dooku (Yoda1 antagonist), GsMTx4 (non-selective mechanosensory channel inhibitor) and L-NAME (nitric oxide synthase inhibitor). In the CC, Yoda1 was also tested in the presence of indomethacin (non-selective COX inhibitor) and tetraethylammonium (TEA, non-selective potassium channel inhibitor). The expression of Piezo1 was confirmed by Western blotting. Our data show that Piezo1 activation leads to the relaxation of the pudendal artery and CC as the chemical activator of Piezo1, Yoda1, relaxed the pudendal artery (47%) and CC (41%). This response was impaired by L-NAME and abolished by Dooku and GsMTx4 in the pudendal artery only. Indomethacin and TEA did not affect the relaxation induced by Yoda1 in the CC. Limited tools to explore this channel prevent further investigation of its underlying mechanisms of action. In conclusion, our data demonstrate that Piezo1 is expressed and induced the relaxation of the pudendal artery and CC. Further studies are necessary to determine its role in penile erection and if erectile dysfunction is associated with Piezo1 deficiency.

## 1 Introduction

The regulation of many cellular and physiological functions such as touch, pain sensation, hearing and blood pressure can be triggered by mechanical forces which will be converted into chemical signals to finally exert their biological response. These stimuli are sensed by several types of mechanosensory channels or receptors located in the membrane of the cells or cellular compartments. Among these mechanosensory channels/receptors, Piezo1 and Piezo2 channels were described in 2010 as mechanically activated cation channels that do not resemble the structure of other ion channels or protein classes (Coste et al., 2010). Since its discovery, Piezo1 channel has been associated with several cellular processes and it was described to be expressed in many different cells, including neural, epithelial, urothelial and endothelial cells, participating in cellular differentiation, proliferation, migration and other cellular processes (Li et al., 2014; Miyamoto et al., 2014; Pathak et al., 2014; Michishita et al., 2016).

In the vasculature, Piezo1 channel is being considered the sensor of shear-stress and it is essential for embryonic development (Ranade et al., 2014). In fact, Piezo1 channel activation induces NO-dependent vasodilation of uterine arteries during late pregnancy. Piezo1 channel is upregulated in the uterine artery, probably to support increased uteroplacental perfusion (John et al., 2018). The importance of the Piezo1 channel in vascular response to shear stress was shown in the endothelial cells of mice lacking Piezo1 channel. These mice exhibited deficiency in stress fiber and cellular orientation in response to shear stress and the embryos missing Piezo1 died at midgestation (Ranade et al., 2014). Moreover, there is evidence of its role in the alignment of endothelial cells according to the blood flow and in the migration of endothelial cells towards the vascular endothelial growth factor (Nourse and Pathak, 2017). Altogether, it is clear that Piezo1 channel plays a role in the function and organization of the vasculature.

Penile erection is a vascular event triggered by a neural stimulus that leads to dilation of the helicine arteries and corpus cavernosum (CC), while the increased blood supply to the penis is reached through the relaxation of the pudendal artery (Calmasini et al., 2019). In both pudendal artery and CC, the relaxation starts with the release of nitric oxide (NO) (Priviero et al., 2007), and vasculogenic erectile dysfunction (ED) is associated with decreased NO bioavailability. To date, no studies have addressed the involvement of Piezo1 channel in the erectile function/dysfunction. Since Piezo1 channel is being considered the sensor of shear stress and it was shown to produce NO-dependent vasodilatation, we hypothesize that Piezo1 plays a role in regulating the vascular mechanisms associated with penile erection. Elucidating the contribution of mechanosensory channels in the pudendal artery and CC may shed light on a better understanding of additional mechanisms contributing to penile erection and unveil potential therapeutic targets for the development of new or adjuvant therapies for the treatment of ED.

## 2 Materials and methods

### 2.1 Animals

Male Wistar rats (11-month-old) were used for this investigation (Envigo, IN, USA). All animal procedures were performed at the University of South Carolina and were conducted in accordance with the Guide for the Care and Use of Laboratory Animals of the National Institutes of Health (NIH) and ethical standards of Institutional Animal Care and Use Committee of the University of South Carolina. The animals were housed one per cage on a 12 h light–dark cycle, with free access to standard chow and water *ad libitum*.

### 2.2 Vascular and cavernosal relaxation studies

Pudendal arteries and CC were rapidly excised and placed in ice-cold physiological solution with the following composition (mM): 130 NaCl, 4.7 KCl, 14.9 NaHCO3, 5.5 dextrose, 1.18 KH2PO4, 1.17 MgSO4, 1.6 CaCl2, and 0.026 EDTA. The pudendal arteries were carefully isolated by dissection away from fat and connective tissue, cut in segments of 2 mm in length and mounted in tissue chambers for isometric tension recording (model 620M; Danish MyoTech, Aarhus, Denmark). The penile tissue was cleaned from connective and adventitial tissues and the fibrous septum separating the corpora cavernosa was opened from its proximal extremity towards the penile shaft. A slit was made in the tunica albuginea along the shaft to obtain 2 strips (11 × 1 × 1 mm) of CC from each animal. Each strip was mounted in a myograph for isometric force recording (model 820M; Danish MyoTech, Aarhus, Denmark). The bathing solution was maintained at 37°C and continuously aerated with 95% O2 and 5% CO2. After stabilization, CC and arterial integrity were assessed by stimulation with 120 mM potassium chloride (KCl). In the pudendal artery, endothelium integrity was assessed by the presence of a relaxation response to acetylcholine (3 μM) during phenylephrine (PE; 1 μM)-induced contraction. The rings that presented relaxation lower than 80% were considered without functional endothelium and were excluded from the study. To investigate the endothelium-dependent relaxation through the activation of Piezo1 channel, the pudendal arteries and CC were contracted with PE (3 and 10 μM, respectively) and concentration-response curves to the chemical activator of Piezo1, Yoda1 (10 μM–100 μM) were obtained in the presence or absence of Dooku (reversible blocker of Yoda1 activity; 1 μM, 30 min) (Evans et al., 2018), GsMTx4 (non-selective mechanosensitive and stretch-activated ion channel inhibitor; 100 nM, 30 min) (Li et al., 2019) and L-NAME (nitric oxide synthase inhibitor; 100 μM, 30 min). In the CC, concentration response curves to Yoda1 were also performed in the presence of indomethacin (non-selective COX inhibitor; 10 μM, 30 min) and TEA (non-selective potassium channels inhibitor; 10 mM, 30 min).

### 2.3 Western blot analysis

Pudendal arteries (pooled from two to three animals to each n) and CC (150 μg and 300 μg, respectively) were homogenized in lysis buffer containing protease inhibitors. Equal amounts of protein were separated by electrophoresis on a 6% polyacrylamide gel and transferred to a nitrocellulose membrane (Sigma-Aldrich). Non-specific binding sites were blocked with 5% skim dry milk in Tris-buffered saline solution with Tween-20 (TBS-T, pH 7.6) for 1 h, at 24°C. Membranes were incubated with the primary antibody Piezo 1 [1:500, pudendal artery; 1:200, CC (NBP-78537, Novus Biologicals)], overnight, at 4°C. On the next day, membranes were removed from primary antibody and washed with TBS-T. Membranes were treated with the secondary antibody for 1 h, at room temperature. The protein bands were visualized using an enhanced chemiluminescence detection system. Results were normalized to the intensity of β-actin protein and are expressed as arbitrary units. Piezo1 antibody was validated in the rat CC in the presence of the blocking peptide (NBP1-78537PEP Novus Biologicals - Data not shown).

### 2.4 Statistical analysis

Data are presented as mean ± standard error mean (SEM) and “n” represents the number of animals used in the experiments, except for pooled samples (see above). Relaxation is expressed as percentage of the decrease in the contraction induced by PE. The relaxation response was analyzed point-by-point for each concentration. Shapiro-Wilk test was used to determine normal distribution. Paired Student’s t-test was performed to compare responses obtained in the same ring or strip. For tests performed in different ring or strip, we used unpaired Student’s t-test. Non-parametric Mann-Whitney test was used for comparisons that did not follow normal distribution. Previous work from our laboratory, as well as power analysis (desired power of 0.80–0.85 with a probability of a Type I error of 0.05), has provided a basis for the number of rats required per experimental group of four to six and this indicates the minimum number needed to generate a statistically significant experimental outcome (*p* < 0.05). We made an effort to reduce the number of animals used in this research, however, after careful consideration of data analysis regarding the scientific merit of the observation. Values of *p* < 0.05 were considered statistically significant.

## 3 Results

**FIGURE 1:**
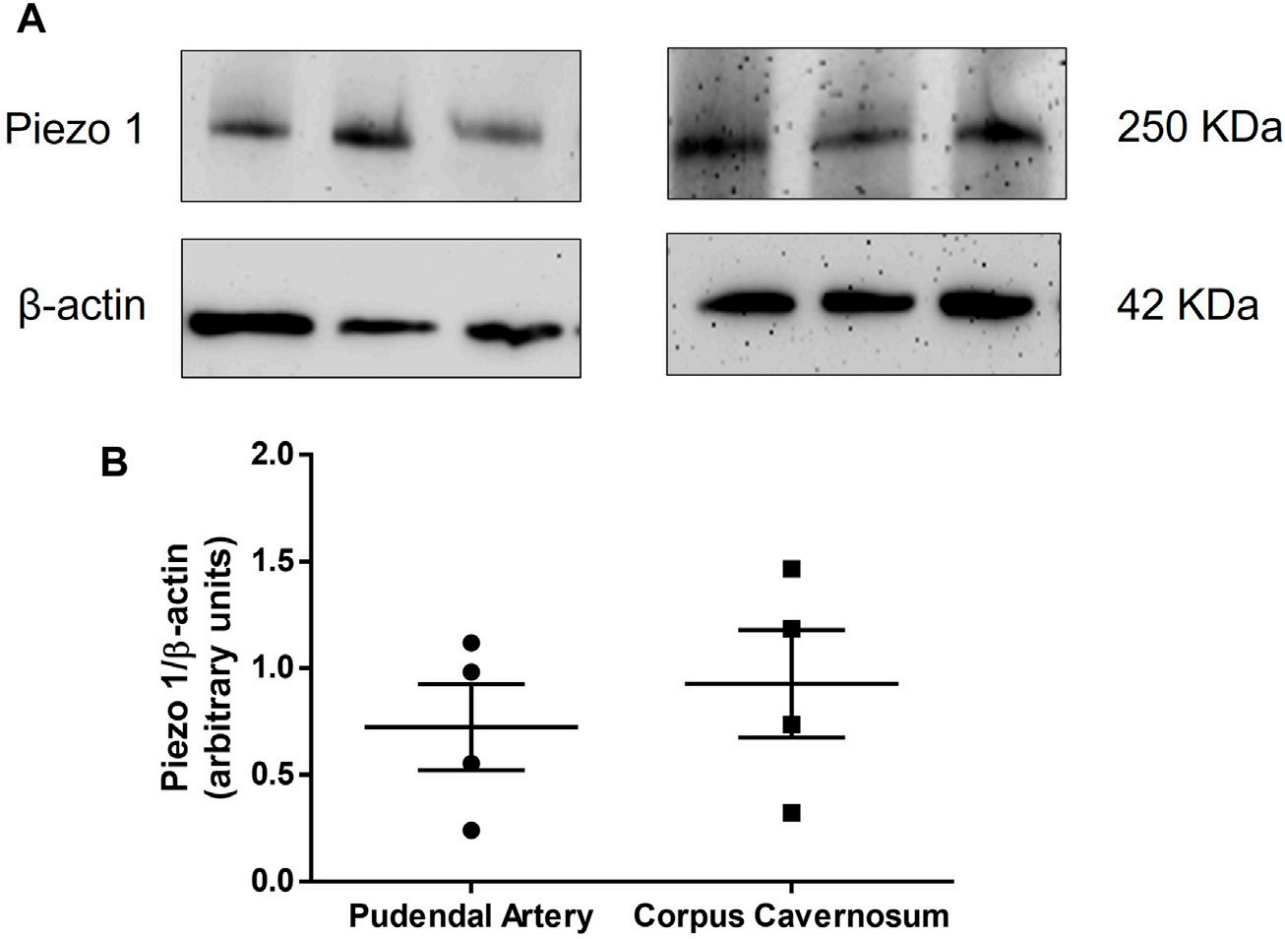
Representative blot **(A)** and protein expression **(B)** of Piezo1 in the pudendal artery (left panel, *n* = 4) and corpus cavernosum (right panel, *n* = 4), respectively.

First, we tested whether Piezo1 channel is expressed in the rat pudendal artery and CC. Figure 1A shows the representative blotting and Figure 1B represents the graph of the expression of Piezo1 in both pudendal artery and CC. It was observed that both pudendal artery and CC strongly express Piezo1 channels. To the best of our knowledge, this is the first study showing that Piezo1 is expressed in erectile tissue.

**FIGURE 2:**
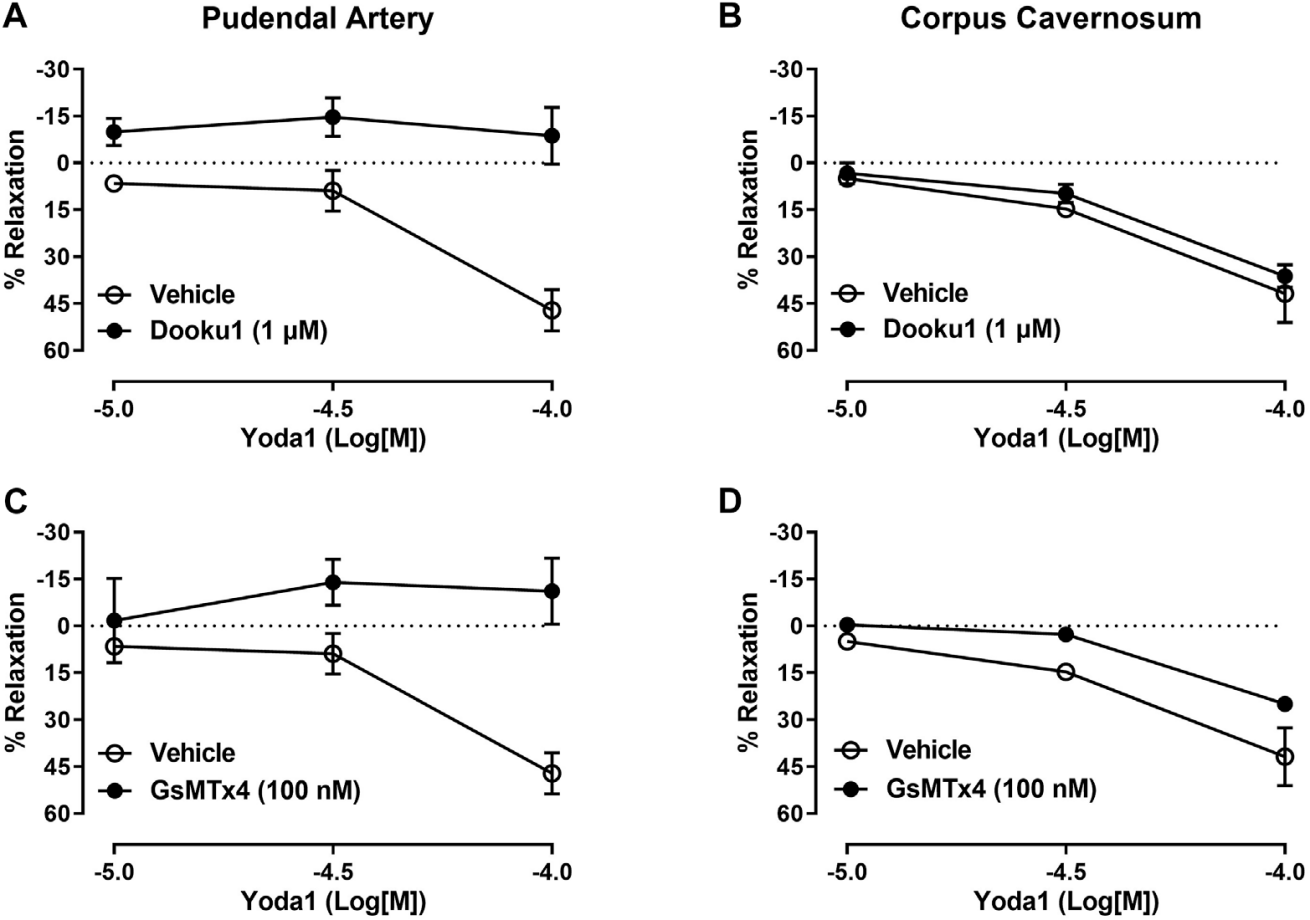
Vascular and cavernosal reactivity to Piezo1 activation induced by Yoda1. Relaxation curves to Yoda1 in the pudendal artery and corpus cavernosum incubated with Dooku **(A, B)**; (*n* = 5), and GsMTx4 **(C, D)**; (*n* = 5). The magnitude of the relaxation induced by Yoda1 was calculated relative to the maximal changes from the contraction produced by PE, which was taken as 100%. Results are presented as mean ± SEM. *p < 0.05 compared to vehicle.

Mechanical activation of the Piezo1 channel is a limiting factor of our study. Therefore, we induced activation of Piezo1 channel using the chemical activator of Piezo1, Yoda1. Yoda1 induced a significant concentration-dependent vasodilation of the pudendal artery (Emax 47%, Figures 2A–C). To demonstrate that Yoda1 is relaxing through Piezo1 channel, we preincubated the pudendal artery with Dooku1 and GsMTx4. Dooku1 is a selective inhibitor of Yoda1 while GsMTx4 is a non-selective cationic channel blocker. Our data showed that both Dooku1 and GsMTx4 completely abolished the vasodilatory response to Yoda1, demonstrating the specificity of Yoda1 for the Piezo1 channel (Figures 2A–C). Similarly, Yoda1 caused concentration-dependent relaxation of the CC (Emax 41%, Figures 2B–D). However, the preincubation with Dooku1 did not cause a significant change in the maximal response to Yoda1 (Emax 36%, Figure 2B), while a slight decrease in the relaxation (Emax 25%, Figure 2D) was seen in the presence GsMTx4.

**FIGURE 3:**
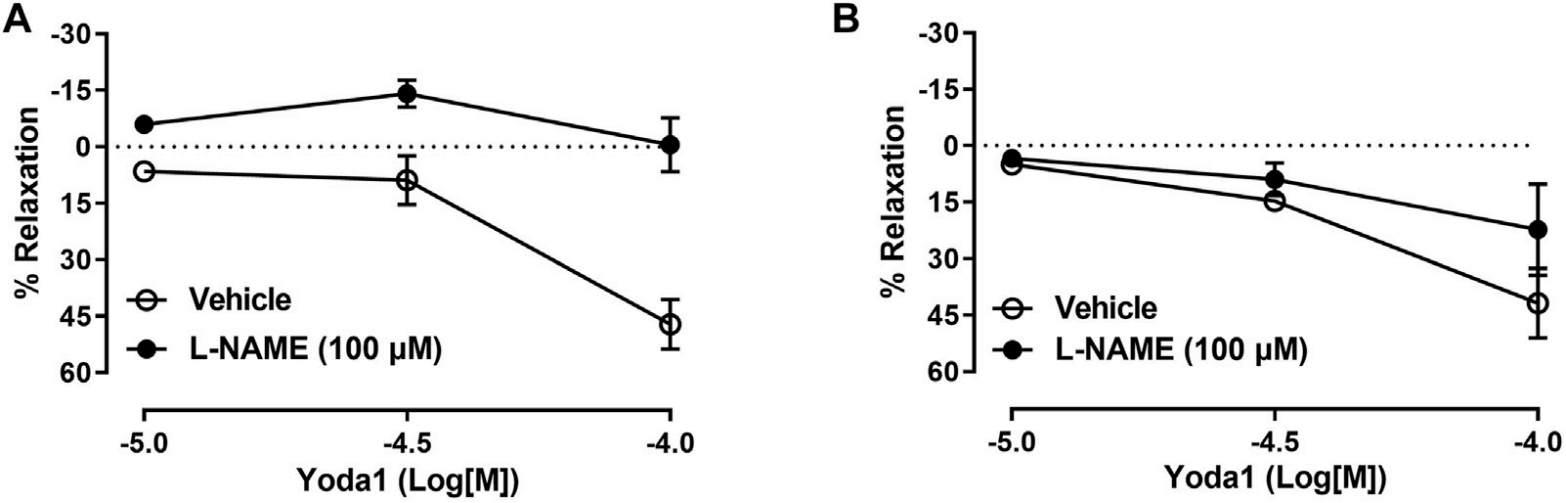
Yoda1-induced relaxation involves NO in pudendal arteries. Incubation with L-NAME abolished the relaxation induced by Yoda1 in the pudendal artery **(A)** but not in the corpus cavernosum **(B)**. Data are mean ± SEM (*n* = 5). *p < 0.05 compared to vehicle.

Because nitric oxide (NO) is considered the main mediator of penile erection, we next tested if the relaxation of the pudendal artery and CC induced by Yoda1 was mediated by NO release. Our data demonstrated that, in the pudendal artery, *in vitro* incubation with L-NAME significantly decreased the relaxation induced by Yoda1, indicating that this response is at least in part mediated by NO (Figure 3A). However, in the CC, it was observed only a slight decrease in the relaxing response induced by Yoda1 in the presence of L-NAME (Emax 22%, Figure 3B).

**FIGURE 4:**
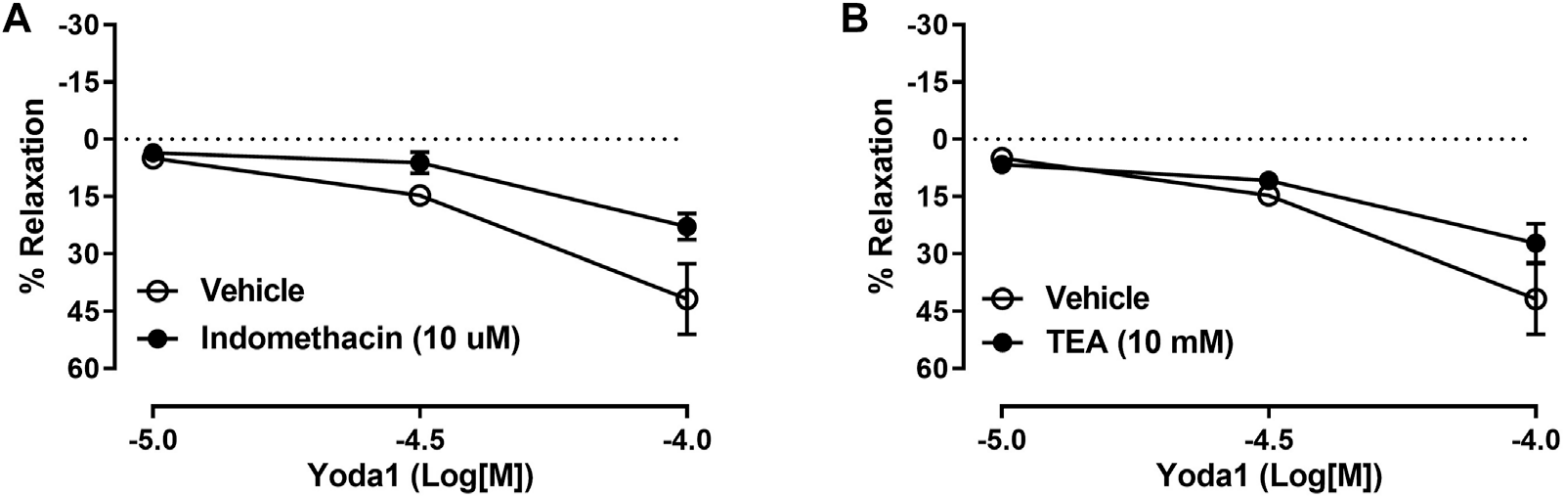
Yoda1-induced relaxation of CC is not affected by COX or potassium channels inhibition. In the CC, incubation with indomethacin **(A)** or TEA **(B)** did not affect the relaxation induced by Yoda1. Data are mean ± SEM (*n* = 4–6). COX (cyclooxygenase); TEA (tetraethylammonium).

To further elucidate the intracellular mechanism of relaxation induced by Piezo1 channel activation in the CC, we performed the curves to Yoda1 in the presence of indomethacin (10 µM) or TEA (10 mM) to test the contribution of COX products or potassium channels activation, respectively, on the relaxation evoked by Yoda1. Figure 4 demonstrates that neither COX inhibition nor potassium channel inhibition changed the relaxation induced by Yoda1 in the CC, ruling out a role for prostaglandins and potassium channel-induced hyperpolarization in Piezo1 activation.

## 4 Discussion

The results presented herein reveal that Piezo1 channels are expressed in the pudendal artery and CC of Wistar rats, and its activation causes a significant relaxation (over 40%) of these tissues. This finding suggests that Piezo1 channel mediated smooth muscle relaxation might be an important mechanism of the initial vasodilatory steps to induce penile erection. Our findings demonstrate that in pudendal arteries, Piezo1 channel activation induces NO-dependent vasodilation, suggesting that Piezo1 may support increased blood supply to the sexual organs during intercourse.

Piezo1 channel has been shown to be a sensitive sensor to shear-stress in vascular endothelial cells. Piezo proteins are components of complex mechanotransducers that include a family of excitatory ion channels directly activated by force (Coste et al., 2010; Ge et al., 2015). The function of Piezo1 ion channel in mechanotransduction has been studied by several groups and it is important for vascular development and maintenance of the structure of the vasculature (Li et al., 2014). The channels are expressed in various mechanically sensitive cell types. The process through by which mechanical forces are translated to physiological responses is very important in the adaptation of the vasculature to flow-induced shear stress and is known as mechanotransduction (Arishe et al., 2020). Further to classic sensory systems, mechanotransduction is involved in several physiological functions, including the regulation of blood flow and vascular tone (Ge et al., 2015; Rode et al., 2017; Beech and Kalli, 2019), essential mechanisms during penile erection.

In this study, we demonstrated that Piezo1 is widely expressed in pudendal artery and CC, two important components in the penile erection, corroborating previous studies that have also identified the presence of Piezo1 channels in other vascular tissues, including aorta, (Li et al., 2014), mesenteric artery (Rode et al., 2017), uterine artery (John et al., 2018), human umbilical vein endothelial cells (HUVEC) (Li et al., 2014) and in a variety of endothelial cells (Li et al., 2014; Ranade et al., 2014; Rode et al., 2017), and they are expressed both at the plasma membrane and in the intracellular compartments (Coste et al., 2010). Physiologically, the activation of Piezo1 in the vasculature increases the concentration of intracellular calcium ([Ca2^+^]i), triggering a specific signaling pathway that results in the activation of eNOS, production of endothelial NO and finally in the vasodilation response (Wu et al., 2017; Beech and Kalli, 2019). Vascular activation of Piezo1 is sufficient to confer shear-stress-evoked iCa2 entry and biological effects on endothelial cells. However, the study of Piezo1 activation is mainly given using a chemical activator, Yoda1, which was screened out of three million compounds, and demonstrated to cause Piezo1 channel opening and calcium ion flow through the membrane of an artificial cell (Syeda et al., 2015). Although little is known regarding the mechanisms of action of Yoda1, a recent study revealed the binding site of Yoda1 in the Piezo1 channel, and described the effects of Yoda1 as a facilitator of conformational changes induced by force (Botello-Smith et al., 2019). Piezo1-specific activator Yoda1 was functionally relevant to induce increases in the concentration of intracellular Ca2+ and concentration-dependent vasodilation in mesenteric arteries (Wang et al., 2016) and uterine arteries (John et al., 2018). Similarly, we observed that Piezo1 activation produces the same effect in pudendal arteries and CC by inducing a significant vasorelaxation in both tissues. In the pudendal artery, the vasorelaxation effect of Yoda1 was abolished after incubation with Dooku1 and GsMTx4, confirming biological activity of Yoda1 and its effect through a mechanosensitive and stretch-activated ion channel such as Piezo1. On the other hand, in the CC, Dooku or GsMTx4 did not affect the response of Yoda1, therefore, more studies using different concentrations of Dooku1 and GsMTx4 as well as intracellular calcium concentration measurements are necessary to better explain the mechanism of relaxation of Yoda1 in the CC. It is important to highlight that Dooku1 is an analogue of Yoda1, exerting its activity by inhibiting Yoda1 effects, and in the absence of Yoda1, Dooku1 is unable to inhibit the constitutive activity of Piezo1 (Evans et al., 2018). Therefore, we acknowledge that the lack of pharmacological tools is a limitation of our study since there is no selective inhibitor of Piezo1 channel. As mentioned above, Dooku1 is an analogue of Yoda1 and selectively antagonizes, in a competitive manner, the activation of Piezo1 induced by Yoda1, the chemical activator of the channel, but not mechanical activation of the channel. However, a recent study demonstrated that Dooku1 caused contraction of the aorta in the absence of the perivascular adipose tissue, raising a possible off target effect for Dooku1 (Miron et al., 2022). On the other hand, GsMTx4, which is a spider venom peptide described before the identification of the Piezo1 channel, (Bowman et al., 2007), was demonstrated by electrophysiological studies to inhibit Piezo1 currents. However, GsMTx4 effects on Piezo1 currents are likely to be an indirect effect of the toxin by modulating the local membrane tension near Piezo1 localization (Suchyna et al., 2000; Suchyna et al., 2004). Therefore, GsMTx4 is known to inhibit cation-permeable mechanosensitive channels, which includes Piezo and TRP channel families, and thus, the effects of GsMTx4 on blocking other TPR channels in our preparations cannot be ruled out (Gnanasambandam et al., 2017).

Despite this, the literature has reported some evidence that corroborate our findings. In the vasculature, Piezo1 depletion or GsMTx4 treatment suppressed shear stress-evoked Ca2+ influx in HUVECs by suppressing the current–voltage relationship of the ionic current reversibly induced by shear stress, abolishing the effects of Piezo1 (Li et al., 2014). Changes in structure and vascular responses were seen in Piezo1 knockout mice. Furthermore, in the mesenteric arteries of these animals, the ability to respond to flow was greatly impaired (Wang et al., 2016). Treatment with GsMTx4 significantly reduced vasodilation to intraluminal flow (John et al., 2018). The functional importance of Piezo1 extends to the control of vascular architecture from the embryonic period, implicating its role in the development and integrity of the circulatory system (Ranade et al., 2014), adequate formation of blood vessels, homeostasis of the volume of red blood cells (Cahalan et al., 2015), regulation of vascular tone, blood flow and blood pressure (Wang et al., 2016; Wu et al., 2017). Integrated and coordinated responses of the vasculature are necessary for adequate stimulation and maintenance of penile erection. Ion channels are also relevant during this process. Upon sexual stimulation, NO production can be achieved in the absence or presence of shear stress (Priviero et al., 2007). In both conditions, eNOS is regulated by the Piezo1 channel. Piezo1 is required for flow-induced ATP release and subsequent phosphorylation and activation of AKT and eNOS (Wang et al., 2016). Furthermore, in the depletion of Piezo1, the expression of eNOS is reduced in HUVECs by abolition of phosphorylation evoked by vascular endothelial growth factor in the residues of eNOS, resulting in dysfunction of eNOS/NO activity and suppression of endothelial cell migration (Li et al., 2014), the main effects associated with ED. Interestingly, low-intensity extracorporeal shockwave therapy is currently used for the treatment of ED despite the unknown underlying mechanisms, and it is believed to act through mechanosensory processes to induce events like neo-angiogenesis, stimulation of progenitor cells, improvement of microcirculation, and nerve regeneration (Sokolakis et al., 2019). It can be speculated therefore, that Piezo1 channel stimulation might be involved in the mechanisms underlying the benefits of the low-intensity extracorporeal shockwave therapy for the erectile function.

Our data demonstrate that treatment with NOS inhibitor suppresses vascular relaxation induced by Piezo1 in the pudendal artery, but not in the CC. It has been established that activation of Piezo1 channel stimulates increases in endothelial NO production leading to endothelium dependent vasorelaxation (Lhomme et al., 2019). Further, NOS inhibition abolishes either nitrergic- or cholinergic-induced CC relaxation in human, rats, mice and rabbits (Mizusawa et al., 2001; Baracat et al., 2003; Teixeira et al., 2004; Tostes et al., 2007). Together, these data suggest that the downstream mechanisms of Piezo1 activation in the CC is independent of NO, which is different from what we observed in the pudendal artery. Indeed, the effect of Piezo1 activation on the vasculature is not limited to the release of NO (John et al., 2018), suggesting that other vasodilators may be involved in this process, such as prostacyclin and endothelium-derived hyperpolarizing factor (Rode et al., 2017). In this context, we performed concentration-response curves to Yoda1 in the presence of the non-selective COX inhibitor, indomethacin, and the non-selective potassium channel inhibitor, TEA. The relaxing response induced by Yoda1 in the rat CC was not affected by either indomethacin or TEA. It is well known that COX activation leads to the synthesis of both contractile and relaxant eicosanoids including the potent vasoconstrictor thromboxane A2 (TXA2) and the vasodilator prostacyclin (Bassiouni et al., 2019). In rat CC, it was demonstrated that indomethacin potentiates the relaxation induced by neuron (electrical field stimulation)- and endothelium (ACh)- derived NO, likely associated with the inhibition of TXA2 synthesis (Bassiouni et al., 2019). However, our experiments failed to demonstrate that COX participates on Yoda1-induced relaxation of the CC, ruling out the involvement of Piezo1 activation in inducing relaxation by either increasing prostacyclin or inhibiting TXA2. Moving forward, we investigated whether EDHF plays a role in the relaxation induced by Piezo1 in the CC. EDHF-induced relaxation of vascular tissues is produced by hyperpolarization in response to stimulation of calcium activated potassium channels (K^+^_Ca_), ATP-sensitive potassium channels and inward rectifier potassium channels (Kir), which are inhibited by the non-selective potassium channel inhibitor, TEA. In rabbit CC, TEA decreases the potency of the relaxation stimulated by ACh (Andre et al., 2003) while in rats, TEA inhibited resveratrol- and L-cysteine induced relaxation of the CC (Dalaklioglu and Ozbey, 2014; Abd Elmoneim et al., 2017). Contrary to these findings, our data show that TEA did not inhibit Yoda1-induced relaxation in rat CC. Therefore, it remains unclear what underlying mechanisms produce relaxation of the CC induced by Piezo1 activation.

In addition, there is evidence demonstrating that in the absence of eNOS activity, Piezo1 activation may lead to smooth muscle depolarization and vasoconstriction when the fluid flow is sufficiently high (Rode et al., 2017; John et al., 2018). For example, Piezo1 activation is necessary to induce vasoconstriction and maintain high blood pressure during physical activity (Rode et al., 2017). However, this effect is not seen during inactivity or when the vasodilator effect and the NO production are important in other circumstances, suggesting that Piezo1 channels act according to the physiological conditions imposed in order to reset cardiovascular homeostasis. This adaptive mechanism may play a central role in the damage caused by the dysfunctional Piezo1 to the physiologic responses that require the availability of NO, including ED. In fact, ED is associated with high blood pressure and other cardiovascular problems, and Piezo1 dysfunction could also contribute to ED. (Wang et al., 2016; Beech and Kalli, 2019). The increase in the bioavailability of NO and the consequent improvement in the relaxation of the CC still represents the main therapeutic approach for ED. Although we are currently reporting a possible role for Piezo1 in the mechanisms of erectile function, several limitations prevent further investigation to determine the role of Piezo1 for this process. To date, Yoda1 is the only chemical compound to induce activation of the Piezo1 channel, and although studies using shear-stress are possible, there is no selective inhibitor of the Piezo1 channel. Dooku is a Yoda1 antagonist and does not inhibit the mechanical activation of Piezo1 channel and GsMTx4 is not a selective inhibitor of this channel. As mentioned above, global Piezo1 knockout animals will die during embryogenic stage (Ranade et al., 2014). Therefore, studies using endothelial or smooth muscle Piezo1 knockout animals would be necessary to further investigate the contribution of this channel to erectile function and a possible association with ED. Thus, the lack of tools to further address the role of Piezo1 for the erectile function and its possible role in the ED is a limitation of our study.

## 5 Conclusion

To the best of our knowledge, this is the first study investigating the involvement of the mechanosensory channel Piezo1 in the mechanisms of penile erection, which involves the relaxation of the pudendal artery and CC. Despite limited tools to address this channel, our findings clearly demonstrated that Piezo1 is widely expressed in the pudendal artery and CC, and its activation induces the relaxation of both tissues. However, while the activation of Piezo1 caused a NO-dependent relaxation of the pudendal artery, Piezo1-induced relaxation of the CC was independent of NO, COX or EDHF. Therefore, more studies are necessary to establish the underlying mechanisms of relaxation evoked by the activation of Piezo1 and its possible role for the development of ED. The continuous advances in the understanding of the physiology of penile erection could further assist in the identification of therapeutic targets for the development of new therapies, especially for patients that do not respond to PDE5 inhibitors or in patients where they are contraindicated (nitrates).

## Data Availability

The raw data supporting the conclusion of this article will be made available by the authors, without undue reservation.
